# The Role of Cumulative Risk Assessment in Decisions about Environmental Justice

**DOI:** 10.3390/ijerph7114037

**Published:** 2010-11-18

**Authors:** Ken Sexton, Stephen H. Linder

**Affiliations:** 1 Department of Epidemiology, Human Genetics, and Environmental Sciences, University of Texas School of Public Health, Brownsville Regional Campus, 80 Fort Brown, Brownsville, TX 78520, USA; 2 Institute for Health Policy, University of Texas School of Public Health, 1200 Herman Pressler, Houston, TX 77030, USA; E-Mail: stephen.h.linder@uth.tmc.edu

**Keywords:** cumulative risk assessment, environmental justice, health disparities, risk assessment, susceptible groups, vulnerable populations

## Abstract

There is strong presumptive evidence that people living in poverty and certain racial and ethnic groups bear a disproportionate burden of environmental health risk. Many have argued that conducting formal assessments of the health risk experienced by affected communities is both unnecessary and counterproductive—that instead of analyzing the situation our efforts should be devoted to fixing obvious problems and rectifying observable wrongs. We contend that formal assessment of cumulative health risks from combined effects of chemical and nonchemical stressors is a valuable tool to aid decision makers in choosing risk management options that are effective, efficient, and equitable. If used properly, cumulative risk assessment need not impair decision makers’ discretion, nor should it be used as an excuse for doing nothing in the face of evident harm. Good policy decisions require more than good intentions; they necessitate analysis of risk-related information along with careful consideration of economic issues, ethical and moral principles, legal precedents, political realities, cultural beliefs, societal values, and bureaucratic impediments. Cumulative risk assessment can provide a systematic and impartial means for informing policy decisions about environmental justice.

## 1. Introduction

Development of viable procedures for evaluating combined threats from cumulative exposure to multiple environmental factors is vital for assessing and ameliorating environmental injustices and associated health disparities [1–6]. Yet procedures to conduct cumulative risk assessments are still under development, and applications to real-world problems are hampered by unavailability of appropriate data, a deficiency of mechanistic understanding, and lack of verified analytical frameworks [1–3]. Currently, it is not apparent to what extent differential cumulative risks from exposure to numerous chemical, biologic, physical, radiologic, and psychosocial agents contribute to higher rates of morbidity and mortality among socioeconomically disadvantaged populations, many of whom are people of color. The challenge is how to develop the necessary models and evaluation methods, acquire crucial knowledge and understanding, and conduct realistic assessments so that risk managers can make informed, scientifically-credible choices about which risks are unacceptable and what, if anything, to do about them.

## 2. Overview of Cumulative Risk Assessment

Cumulative risk assessment is a science-policy tool for organizing and analyzing information to examine, characterize, and possibly quantify combined adverse effects from chemical (e.g., benzene, mercury, polycyclic aromatic hydrocarbons) and nonchemical (e.g., pollen, noise, microwave radiation, unsafe neighborhoods, unemployment) stressors in the environment [2,3]. Conventional risk assessments have traditionally focused almost exclusively on single chemicals, specific health endpoints, individual sources or source categories, a particular environmental medium (e.g., air, water, food, soil), and a single exposure pathway and route; cumulative risk assessment is a more expansive application that encompasses multiple stressors, endpoints, sources, environmental media, and pathways and routes of exposure. Recent summaries of the cumulative risk literature [1–3] suggest an emerging consensus on a core set of differences that distinguish cumulative assessments from conventional ones. Cumulative assessment:

- involves evaluation of collective health effects of multiple stressors [as opposed to individual effects of a single stressor];
- broadens the spectrum of environmental agents being appraised to include psychological (e.g., residential crowding) and sociological (e.g., racial discrimination) stressors [not just chemicals];
- focuses on population-based or location-based assessments of real-world cumulative exposures experienced by actual people [most conventional assessments entail source-based assessments of hypothetical people and theoretical exposures].

In practice, cumulative assessments have introduced a number of other differences as well. They have:

- incorporated the concept of vulnerability (*i.e.*, differential a. biological susceptibility, b. exposure, c. preparedness to withstand stressor effects, and d. ability to recover from stressor effects)
  into the assessment explicitly [rather than treating it implicitly as is done in most conventional assessments];
- recognized that the details (e.g., co-exposure to multiple agents, timing of exposure) and history (e.g., continuous *versus* intermittent, simultaneous *versus* sequential) of exposure to multiple stressors may be important for predicting risk [conventional assessments typically assume adverse effects are related solely to a combination of duration and intensity];
- taken account of background exposures (*i.e.*, combined exposure to toxicologically relevant environmental stressors that are not necessarily the focus of the assessment), which may contribute to the cumulative risk under consideration [not normally evaluated as part of conventional risk assessments];
- provided for the possibility of a semi-quantitative or qualitative analysis/result, depending on the circumstances [in contrast to most previous assessments, which are quantitative].

Although cumulative risk assessment aims to answer important and formerly unaddressed questions about combined risk burdens and disproportionate adverse health effects, it is more complex theoretically, methodologically, and computationally than traditional single-chemical, source-oriented assessments [1–3]. Consequently, conceptual models, theoretical frameworks, analytical procedures, and assessment methods are still under development, and relatively few cumulative risk assessments have been conducted in the field [1–3]. In 2003, the U.S. Environmental Protection Agency (EPA) [3] published a framework for cumulative risk assessment that provided a conceptual structure for identifying the fundamental elements and basic principles of an organized process for conducting and evaluating cumulative risk. The aim was to propose a flexible structure that encouraged dialogue on theoretical issues, technical matters, key definitions, and implementation challenges. The EPA [3] described the framework as an information document that identified important features of cumulative risk assessment “whether or not the methods or data currently exist to adequately analyze or evaluate those aspects of the assessment”.

**Figure 1:**
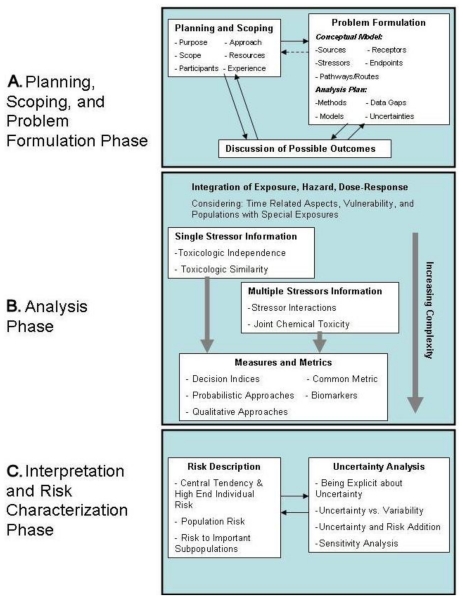
The U.S. Environmental Protection Agency’s framework for cumulative risk assessment, from [3].

The EPA cumulative risk assessment framework, as shown in Figure 1, describes three interrelated and generally sequential phases: Phase 1—planning, scoping, and problem formulation; Phase 2—information and data analysis; and Phase 3—interpretation and risk characterization. In the first phase, risk assessors, risk managers, and interested stakeholders work together to determine the goals, scope, and focus of the assessment. The products of this phase are a conceptual model that identifies stressors, effects, and stressor-effect relationships and an analysis plan specifying the data needed, the approach to be taken, and the types of results expected. The second phase involves technical/scientific activities such as developing exposure profiles, examining the nature and extent of interactions among stressors, estimating risks, and discussing related issues of variability and uncertainty. The products of phase two are estimates of the cumulative risk from exposure to the multiple stressors of interest, and of the variability and uncertainty associated with the predicted risk. In the third phase, risk estimates are explained and their significance described in terms of reliability and confidence placed in the calculated values. In addition, effects of key assumptions are detailed, the uncertainties involved are delineated, and a determination is made as to whether the assessment met the goals and objectives set forth in phase 1 [2].

In 2009, the National Research Council (NRC) [1] published a report, *Science and Decisions: Advancing Risk Assessment* (also known as the Silver Book), evaluating current risk assessment and risk management practices in environmental health. This report is an update of the NRC’s landmark study [7] *Risk Assessment in the Federal Government: Managing the Process* (also known as the Red Book), which in 1983 established the risk assessment—risk management paradigm still in use today. In its most recent report, the NRC noted that there is increasing concern among stakeholders (especially communities affected by obvious sources of environmental pollution) that past risk assessments have been overly narrow, and that they do not capture the cumulative risks from exposure to multiple chemical and nonchemical stressors, nor do they incorporate other factors that could influence vulnerability. The NRC opined that unless cumulative risks are taken into account, risk assessment might become irrelevant in many decision contexts and, furthermore, that continued application of restricted assessments that ignore combined health effects from chemical and nonchemical stressors could exacerbate longstanding credibility and communication gaps between risk assessors and many stakeholders. To enhance the utility of cumulative risk assessment for risk-based decision making, the NRC [1] recommended that EPA do the following:

- maintain the core definitional components of cumulative risk assessment from the 2003 framework document;
- revise the structure for risk-based decision making to focus more on discriminating among risk management options, and thereby narrow the scope of cumulative risk assessments to those stressors that would either be influenced by practical risk management options or modify the risks of other stressors influenced by risk management options;
- explicitly define and maintain conceptual distinctions among cumulative risk assessment, cumulative impact assessment, and community-based risk assessment;
- develop, in the near term, databases and default approaches to incorporate key nonchemical stressors into cumulative risk assessments in the absence of population-specific data;
- fund research and develop internal capacity related to interactions between chemical and nonchemical stressors;
- focus on developing guidelines and methods for simplified analytic tools that allow for screening-level cumulative risk assessments, and which could provide tools for use by communities and other stakeholders.

Most of the NRC’s recommendations address the need for a more versatile tool to support policy decisions on the inequitable distribution of environmental hazards, emphasizing the necessity of extending current approaches with new data and new frameworks and models. The move toward cumulative assessment of combined risks is in line with both expert recommendations [2,3] and stated concerns of environmental justice advocates [6].

## 3. Risk Assessment—Problem or Solution?

Risk assessment is not embraced by everyone as a helpful decision-making tool. In fact, opinions about the value of risk-based decision making fall generally into one of two antithetical domains: those who believe risk assessment is part of the problem and those who believe it is part of the solution [8,9]. People who see it as part of the problem tend to view risk assessment as an ethically suspect, resource-intensive, elitist, never-ending process used by those in power to maintain the status quo [10–15]. To them, risk assessment provides a convenient excuse to avoid the problem, exploits the veil of expert judgment to exclude public values, functions to ignore or trivialize certain hazards, and goes astray by placing the burden of proof on the public rather than on the proponent of an activity, substance, or technology [8]. They argue that “the proof is in the pudding” as demonstrated by the fact that risk assessment has consistently failed to protect public health and environmental quality in poor, minority communities. The detractors’ viewpoint is illustrated by two representative quotations.

“Risk assessment methodology currently incorporates numerous informational biases that may disproportionately affect poor communities and communities of color. Specifically, risk assessments generally fail to observe those health effects that result from above-average exposure, from exposure to multiple chemicals, and from interactions of chemicals. Similarly, risk assessments generally fail to observe susceptibility differences as a function of race and income [11].”

“From an environmental justice perspective, the rush to embrace and expand the use of quantitative risk assessment is not justified. As currently structured and as proposed for more widespread use, the process does not offer a safe haven from distributional inequities or from the dominating influences of resources and political power on environmental decision making [12].”

Proponents of risk assessment, on the other hand, assert that it is an essential decision-making tool for identifying, documenting, and resolving issues of environmental justice [1–3,6,8,9,16,17]. They declare that risk assessment provides a unifying conceptual framework and a common language for addressing environmental justice concerns. Furthermore, they argue that it serves as an indispensable methodology for rational estimation and comparison of environmental health risks, which benefits all members of society [8,9]. Two quotes from risk assessment supporters exemplify this point of view.

“While some advocates of environmental justice are wary of risk analysis … we see comparative risk analysis as a promising ally for those concerned that insufficient resources have been dedicated to improving the welfare of low-income and minority populations. We suspect that many of the risks in America that would score high in risk-ranking exercises are indeed ones that strike poor people and disadvantaged citizenry with disproportionate frequency [18] …”

“The process of risk assessment has been used to help us understand and address a wide variety of hazards and has been instrumental to the U.S. Environmental Protection Agency, other federal and state agencies, industry, the academic community, and others in evaluating public-health and environmental concerns. From protecting air and water to ensuring the safety of food, drugs, and consumer products such as toys, risk assessment is an important public-policy tool for informing regulatory and technical decisions, setting priorities among research needs, and developing approaches for considering the costs and benefits of regulatory policies [1].”

As summarized below, criticism of risk assessment can be divided into eight recurring and overlapping prototypical critiques, to which proponents of risk assessment typically respond with standard rebuttals [8]. The important point is that these critiques of conventional risk assessment can be seen to foreshadow subsequent developments in cumulative designs that are slowly supplanting conventional approaches in many community-based applications.

- Ethical Critique—Risk-based approaches are unethical because they fail to safeguard human health and environmental resources adequately. Response—To the contrary, the evidence indicates that risk-based approaches have been largely successful in protecting people and environmental quality, and that their effectiveness continues to improve over time.
- Paradigm Critique—The “precautionary principle” should replace the traditional risk assessment—risk management paradigm because it places the burden of proof on proponents to show that potentially hazardous activities, substances, and technologies represent acceptable risks, instead of requiring the public to demonstrate that risks are unacceptable. Response—Decisions about who should bear the burden of proof are value-based policy choices reflecting societal judgments. Moreover, either explicit or implicit evaluation of risk is an intrinsic component of both the precautionary principle and risk-based decision making.
- Empirical Critique—Valid risk assessments are precluded in most cases by large scientific uncertainties, which derive from both a scarcity of data and limitations on our ability to interpret existing information. Response—Formalized risk assessment provides a valuable framework for organizing and analyzing available scientific information and for identifying data gaps and methodological shortcomings. It also affords a formalized procedure to recognize, examine, and discuss crucial scientific uncertainties likely to affect risk estimates.
- Obstructionist Critique—The difficulties inherent in establishing causality and meeting the data requirements of quantitative risk assessment needlessly bog down the decision-making process, frequently leading to “paralysis by analysis.” Response —Policy decisions about protecting public health need not and should not be delayed by a contrived and superfluous obligation to complete a comprehensive and quantitative assessment of risk. If the stakes are high enough, decision makers have a responsibility to take precautionary action when public health and/or environmental quality are threatened with serious and irreversible harm even if some cause-effect relationships are not fully established scientifically. In these circumstances, an unfinished risk assessment is never an adequate excuse for doing nothing.
- Methodological Critique—By focusing inappropriately on a single dimension of risk (probability X severity), quantitative risk assessment ignores other aspects, like fear, dread, and outrage, which are likely to be more important. Response—Expert evaluation of the likelihood and seriousness of harm establishes a scientifically-credible underpinning for sound decision making, and it does not preclude or impede consideration of other relevant factors, including public perceptions and values.
- Political Critique—Despite claims that it produces more rational, science-based decisions, risk assessment is actually used as a smokescreen by those who seek to ignore or trivialize certain risks. Response—Most proponents and practitioners of risk assessment have no vested interest in the outcome, and defend its use because they believe proper application leads directly to more informed and more reasonable environmental decisions.
- Procedural Critique—Whether or not it is more rational, the process of relying exclusively on expert judgment to evaluate risks is undemocratic because citizens and communities have a right to participate in decisions that affect their health and well-being. Response—It does not have to be one way or the other. An integrated approach, which involves the public along with experts in identifying and evaluating risk, is emerging as a middle-of-the-road alternative.
- Irrelevance Critique—Conventional risk assessment has focused narrowly on individual (primarily chemical) risks, emphasizing single health outcomes, sources, pathways and routes of exposure; but people in the real world are exposed to complex mixtures of environmental hazards (including nonchemical stressors) from diverse sources via multiple pathways/routes, which means the emphasis should be on assessing the overall effect of all of these factors. Response—The potential significance of combined health effects from mixtures of environmental agents is well known, and efforts are underway in the U.S. and Europe to develop methods and procedures for assessing cumulative risks from combinations of hazards encountered by people during their everyday activities; several estimation methods are already available for joint risks from chemicals with a common mechanism of toxicity or that damage the same target organ.

Environmentalists, environmental justice champions, community activists, many concerned citizens, and some academics are longtime critics of risk assessment [10–15], whereas business people, regulatory officials, numerous scientific organizations, and many environmental health scientists are stanch defenders [1,7,16,17]. The modifications introduced by cumulative approaches are likely to recast this debate by shifting the focus of disputes away from the technique itself and toward the costs and equity of the results.

## 4. Putting Risk Assessment Principles into Practice

The use of cumulative risk assessment as a tool for regulatory decision making began in 1986 when EPA issued guidelines [19] for evaluating health risks from chemical mixtures, which were updated in 2000 [20] and expanded in 2006 [21]. Over the past 25 years, processes and procedures to conduct cumulative risk assessment have gradually evolved, and the scope has expanded to include both chemical and nonchemical stressors. According to the NRC [1], the EPA [2,3], and the National Environmental Justice Advisory Council [6], implementation of cumulative risk assessment is meant to broaden the extent of scientific analysis so as to incorporate psychological and sociological sources of stress (even if quantitative methods are not available), thereby making assessments more (a) realistic in the sense of embodying actual, real-life situations and circumstances, (b) reliable as input parameters to risk management decisions, (c) relevant to the problems confronting elected officials and regulatory decision makers, and (d) responsive to stakeholder concerns.

In the past, the vast majority of cumulative risk assessments have examined mixtures of chemicals with either similar mechanisms of toxic action, such as drinking water disinfection byproducts, polychlorinated biphenyls or PCBs, and organophosphate pesticides, or similar health endpoints, such as coke oven emissions, environmental tobacco smoke, and diesel exhaust. Although cumulative risk assessment has been applied in an increasing number of contexts over the past decade, and despite the fact that the definition explicitly includes nonchemical stressors, no cumulative risk assessments by EPA have formally incorporated psychosocial stressors like discrimination and poverty [1]. This situation is changing, however, with the availability of new methods and tools [21–23], as well as more rigorous theoretical paradigms and analytical frameworks [2,3,6,24–30].

**Figure 2:**
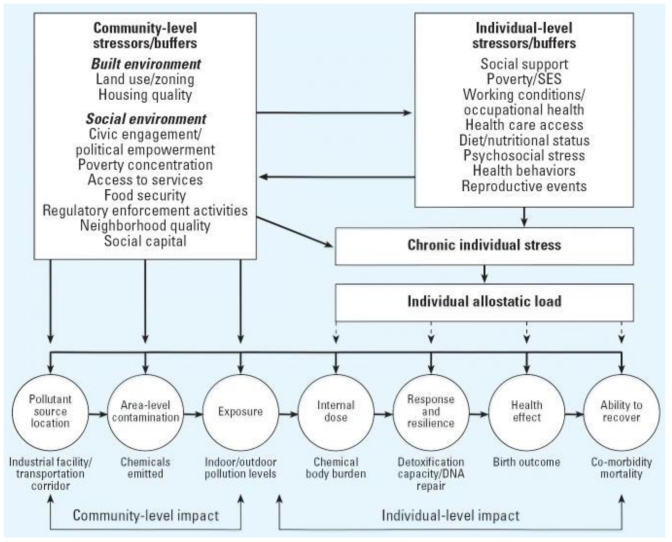
Conceptual model depicting combined health effects from exposure to chemical and nonchemical stressors, from [25].

An example of a conceptual model [25] that postulates causal factors and pathways for cumulative health effects from exposure to chemical and nonchemical stressors is shown in Figure 2. It uses an exposure-stress-effect framework to hypothesize that important stressors and buffers function at both the community-level (e.g., built environment, social environment) and individual-level (e.g., social support, health behaviors), and posits that a combination of chemical and nonchemical stressors contributes to chronic individual stress. Increased chronic stress can then cause an increase in allostatic load, which is defined as the cumulative effects over time of adaptive processes to acute stress. A high allostatic load can lead to illness or injury through wear and tear on the body and brain from being chronically “stressed out.” This model uses the concept of allostatic load as a mechanism to link stress-induced biological responses to observed health disparities, thereby providing a viable method for incorporating psychosocial stressors into cumulative risk assessments. It should be noted that this framework represents one of a number of candidates rather than a consensus version, and that it incorporates nomenclature from the social and behavioral sciences, which is not necessarily consistent with mainstream studies in exposure analysis and environmental health.

Because there is currently no scientific consensus concerning appropriate conceptual models for structuring cumulative risk assessments, a variety of methods and approaches have been proposed [29,30]. Among the assortment of contemporary techniques are: the Cumulative Environmental Hazard Inequality Index (CEHII) developed by scientists at the University of California, Berkeley [31], which creates an index summarizing racial, ethnic, and socioeconomic inequalities from cumulative effects of multiple environmental hazards; the World Health Organization’s [32] Urban Health Equity Assessment and Response Tool (Urban HEART), which identifies and analyzes health disparities between people living in different sections of a city or between different socioeconomic groups within or across cities; the EPA’s [33] Community-Focused Exposure and Risk Screening Tool (C-FERST), which is a web-based tool with links to EPA information and methods that is being developed for use by communities to identify and prioritize cumulative health risks; and the Environmental Justice Strategic Enforcement Screening Tool (EJSEAT) created by EPA’s Office of Enforcement and Compliance Assistance [34]. Most of these techniques work with sets of empirical indicators that serve as proxies for exposures to a range of chemical and non-chemical stressors, which, when considered as composites, provide a short-hand method for cumulating effects.

The EJSEAT is intended to provide for consistent identification of geospatial areas with potentially disproportionately high burdens of harmful environmental factors or features. It is composed of 18 empirical measures divided into four categories (environmental—6 measures, human health—2 measures, compliance—4 measures, and socio-demographic—6 measures). A simple algorithm is used to identify areas with elevated EJSEAT scores, which indicate a high prevalence of undesirable or hazardous conditions. Values for each of the 18 indicators are derived from publicly available databases for every one of the approximately 65,000 census tracts in the United States. All measures within a category are normalized and then combined into a single category score. Each of the four category scores are themselves normalized and then averaged to produce a composite EJSEAT score. The raw EJSEAT score is normalized and used as the basis for comparing census tracts and identifying those representing the most serious cases of environmental injustice.

Today, there is a growing need for verified frameworks and practical methods to assess cumulative health risks in the context of health disparities and environmental injustices. The problem with identifying and testing an appropriate framework is that formalized evaluation of combined health effects from chemical and nonchemical stressors, which necessarily includes consideration of background exposures and disease processes along with other aspects of vulnerability, can quickly become analytically intractable because of either computation requirements or scarcity of essential data [1,35]. Nevertheless, applications of cumulative risk assessment, even if they are incomplete or flawed, focus attention on why and how differential cumulative exposures occur, the conditions under which they give rise to divergent health risks, and the mechanisms by which they translate into health disparities.

## 5. Conclusions

Cumulative risk assessment has the potential to answer some, but not all of the questions posed by affected communities and groups [1]. For example, assessment of cumulative health risk can, in principle, answer questions like “What are the sources of pollution in our community that may be causing or contributing to observed health effects?” or “Which intervention strategies are most likely to improve environmental health in our community?” But broader questions like “Should another industrial plant or roadway be added to our already polluted community?” or “Should mitigation activities be undertaken because our poor, minority neighborhood bears a greater burden of locally unwanted land uses than affluent, white neighborhoods?” reflect fundamental concerns about what kind of society we want to live in. In these instances, cumulative risk assessment can only provide limited information on one aspect of a complicated policy question that requires decision makers to weigh a diversity of factors including, not only risk-related information, but also economic issues, legal precedents, political realities, bureaucratic impediments, ethical and moral principles, societal values, and cultural beliefs and attitudes.

Risk-based decisions involve elements of both (a) science, including activities such as research and development, monitoring and data collection, review and interpretation of technical investigations, and evaluation of health and environmental risks, and (b) policy, used here to mean value-driven risk-management decisions about the acceptability of estimated risks and the appropriate tradeoffs between costs and benefits associated with preventing or reducing those risks deemed unacceptable. The interface between science and policy is referred to as “science policy,” which has two complementary meanings: the use of science to make judgments about the formulation and implementation of policy (e.g., quantitative risk assessment) and the development of policy specifically for science (e.g., setting research directions and priorities). Science policy necessarily functions in a realm where scientific knowledge and understanding are incomplete; consequently, judgments, inferences, and extrapolations are inherent components of virtually all science-policy activities [36].

Cumulative risk assessment is, by definition, a science-policy mechanism that unavoidably incorporates science-based assumptions and expert opinions in order to estimate combined health risks from exposure to multiple environmental stressors. The shortcomings inherent in this approach are apparent to advocates and critics alike, but its value lies in the establishment of a formal structure for organizing and analyzing scientific information about combined health effects from chemical and nonchemical stressors. If performed correctly, cumulative risk assessment does more than just generate reliable risk estimates; it also makes explicit the critical underlying assumptions and associated scientific uncertainties. What is more, it provides a vehicle for framing important risk-related issues and structuring the debate about how to address them. The main point to remember is that cumulative risk assessment is a tool to aid decision makers—not a hard and fast rule that prevents them from using their discretion.

Because there is so much at stake, decisions about environmental justice, whether they are risk-based or not, will always be contentious. Cumulative risk assessment can promote policy choices that are more amicable and consensual by providing a systematic and impartial process for identifying and characterizing combined risks. We don’t need a quantitative risk assessment to tell us what we already know; namely, that environmental health risks are likely to be higher for socioeconomically disadvantaged populations. But we do need cumulative risk assessment to understand which environmental mixtures of chemical and nonchemical stressors are most critical from a public health perspective, determine the nature and magnitude of relevant cumulative exposures for the population of interest, and delineate key interaction mechanisms and related health consequences for the constituents of high-priority mixtures. This type of risk-based information is the scientific bedrock upon which informed decisions about environmental justice must be based in order to ensure that selected risk-management options are effective (e.g., mitigation measures achieve stated goals), efficient (e.g., objectives are attained using low-cost approaches), and equitable (e.g., vulnerable populations are protected adequately).
